# Physiological characterization of a novel PPAR pan agonist, 2-(4-(5,6-methylenedioxybenzo[*d*]thiazol-2-yl)-2-methylphenoxy)-2-methylpropanoic acid (MHY2013)

**DOI:** 10.18632/oncotarget.14818

**Published:** 2017-01-25

**Authors:** Hye Jin An, Bonggi Lee, Dae Hyun Kim, Eun Kyeong Lee, Ki Wung Chung, Min Hi Park, Hyoung Oh Jeong, Sung Min Kim, Kyoung Mi Moon, Ye Ra Kim, Seong Jin Kim, Hwi Young Yun, Pusoon Chun, Byung Pal Yu, Hyung Ryong Moon, Hae Young Chung

**Affiliations:** ^1^ College of Pharmacy, Pusan National University, Busan 46241, Republic of Korea; ^2^ Molecular Inflammation Research Center for Aging Intervention (MRCA), Pusan National University, Busan 46241, Republic of Korea; ^3^ College of Pharmacy, Inje University, Gyeongsangnam-do 50834, Republic of Korea; ^4^ Department of Physiology, The University of Texas Health Science Center at San Antonio, San Antonio, TX 78229-3900, USA; ^5^ Korean Medicine (KM)-Application Center, Korea Institute of Oriental Medicine (KIOM), Daegu 41062, Republic of Korea

**Keywords:** MHY2013, PPAR pan agonist, metabolic syndrome, FGF21, adiponectin

## Abstract

Recently, agonists targeting multiple peroxisome proliferator-activated receptors (PPARs) have been developed to improve metabolic disorders and minimize the side effects of selective PPAR agonists such as weight gain and dyslipidemia. We newly synthesized six 2-methyl-2-(*o*-tolyloxy)propanoic acid derivatives based on the structure of a well-known PPAR pan agonist, bezafibrate. Of six compounds, MHY2013 was screened as the strongest activator of three PPAR subtypes based on protein docking simulation and luciferase assays. When treated orally in db/db mice, MHY2013 ameliorated obesity-induced insulin resistance, dyslipidemia, and hepatic steatosis without changes of the body weight and levels of liver and kidney injury markers. MHY2013 decreased the serum triglyceride and fatty acid levels, which is associated with an increase in fatty acid oxidation signaling in the liver and thermogenic signaling on white adipose tissue, respectively. Furthermore, MHY2013 markedly increased serum levels of insulin-sensitizing hormones including fibroblast growth factor 21 (FGF21) and adiponectin. In conclusion, this study suggests that, MHY2013 is a novel PPAR pan agonist that improves obesity-induced insulin resistance, dyslipidemia and hepatic steatosis and elevates insulin-sensitizing hormones in the blood.

## INTRODUCTION

Each PPAR subtype plays a pivotal role in regulating tissue metabolism in hormone-dependent and independent manners [1]. PPARα is mainly expressed in the liver, muscle, and heart, stimulating β-oxidation and energy expenditure [2–4], whereas PPARγ is highly expressed in adipose tissue where it regulates adipocyte differentiation and insulin sensitivity [2, 3]. Although biological functions of the ubiquitously expressed PPARβ/δ need to be elucidated, diverse studies have suggested its role in regulating lipid homeostasis and energy utilization. The PPARs are among the most remarkable targets for the treatment of metabolic syndrome [5]. Consequently, PPAR subtype agonists are in clinical use for metabolic diseases, including type 2 diabetes, cardiovascular diseases, etc. [6]. However, unresolved issues exist for each PPAR subtype agonist. Fibrate-type drugs targeting PPARα are associated with hepatic toxicity, cholelithiasis, and myopathy, which limit their wider application in patients [7]. In the case of PPARγ selectively agonistic glitazone-type drugs, side effects such as dyslipidemia, heart failure, and weight gain have been reported in clinical stages [7]. Among those, rosiglitazone was even withdrawn in Europe, and its use has been restricted in the USA. Clinical and animal studies have demonstrated beneficial effects of PPAR dual and pan agonists, with fewer side effects compared to selective PPAR agonistic drugs, possibly by providing complementary effects [8]. Hence, development of PPAR dual/pan agonistic drugs capable of balanced activation of each PPAR subtype may offer a fascinating therapeutic option. In line with this, we newly synthesized and screened six 2-methyl-2-(*o*-tolyloxy)propanoic acid derivatives as candidates for PPAR pan agonists and examined their effects on insulin resistance and dyslipidemia in genetically obese db/db mice. Our data showed that MHY2013 was the strongest PPAR pan agonist among them. MHY2013 elevated blood FGF21 and adiponectin and increased adipose tissue browning, thereby contributing to improved obesity-induced insulin resistance, hepatic steatosis, and dyslipidemia without affecting body weight. Our study provides a molecular rationale for further development of MHY2013 as treatment of metabolic disorders.

## RESULTS

### MHY2013 is a potent PPAR pan agonist *in vitro*

**Figure 1:**
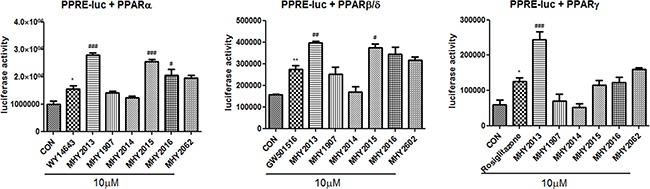
MHY2013 is the strongest PPAR pan agonist among six newly synthesized compounds For luciferase assays to screen of six newly synthesized compounds for PPAR pan agonist, the 3X-PPRE-TK-LUC plasmid and respective PPAR subtype expression vectors were transfected in YPEN-1 cells. Twenty-four hours after the transfection, the cells were treated with the indicated new compounds or respective PPAR agonists (WY14643, GW501516 or rosiglitazone) for 5 h. The data are shown as the mean ± SEM (n = 4) luciferase activity, considered to represent the PPAR transcriptional activity. *, *p* < 0.05 vs. DMSO control (CON); **, *p* < 0.01 vs. CON; ***, *p* < 0.001 vs. CON; #, *p* < 0.05 vs. WY14643, GW501516 or rosiglitazone; ##, *p* < 0.01 vs. WY14643, GW501516 or rosiglitazone; ###, *p* < 0.001 vs. WY14643, GW501516 or rosiglitazone.

To search for powerful PPAR pan agonists, we newly synthesized six 2-methyl-2-(*o*-tolyloxy)propanoic acid derivatives based on the structure of the well-known PPAR pan agonist bezafibrate (Supplementary Method 1 and Supplementary Table 1). The PPRE-luciferase assays for the three PPAR subtypes were performed using YPEN-1 cells to screen for the strongest PPAR pan agonist. Among the six derivatives, MHY2013 had the highest luciferase activities for all PPAR subtypes, and its effect on each PPAR subtype was comparable to that of each specific PPAR subtype agonist (PPARα: WY14643, PPARβ/δ: GW501516, and PPARγ: rosiglitazone) (Figure 1). In addition, MHY2013 dose-dependently increased luciferase activities for the three PPAR subtypes, suggesting that the compound strongly activates all PPAR subtypes (Supplementary Figure 1).

### MHY2013 can directly bind to three PPAR subtypes

**Figure 2:**
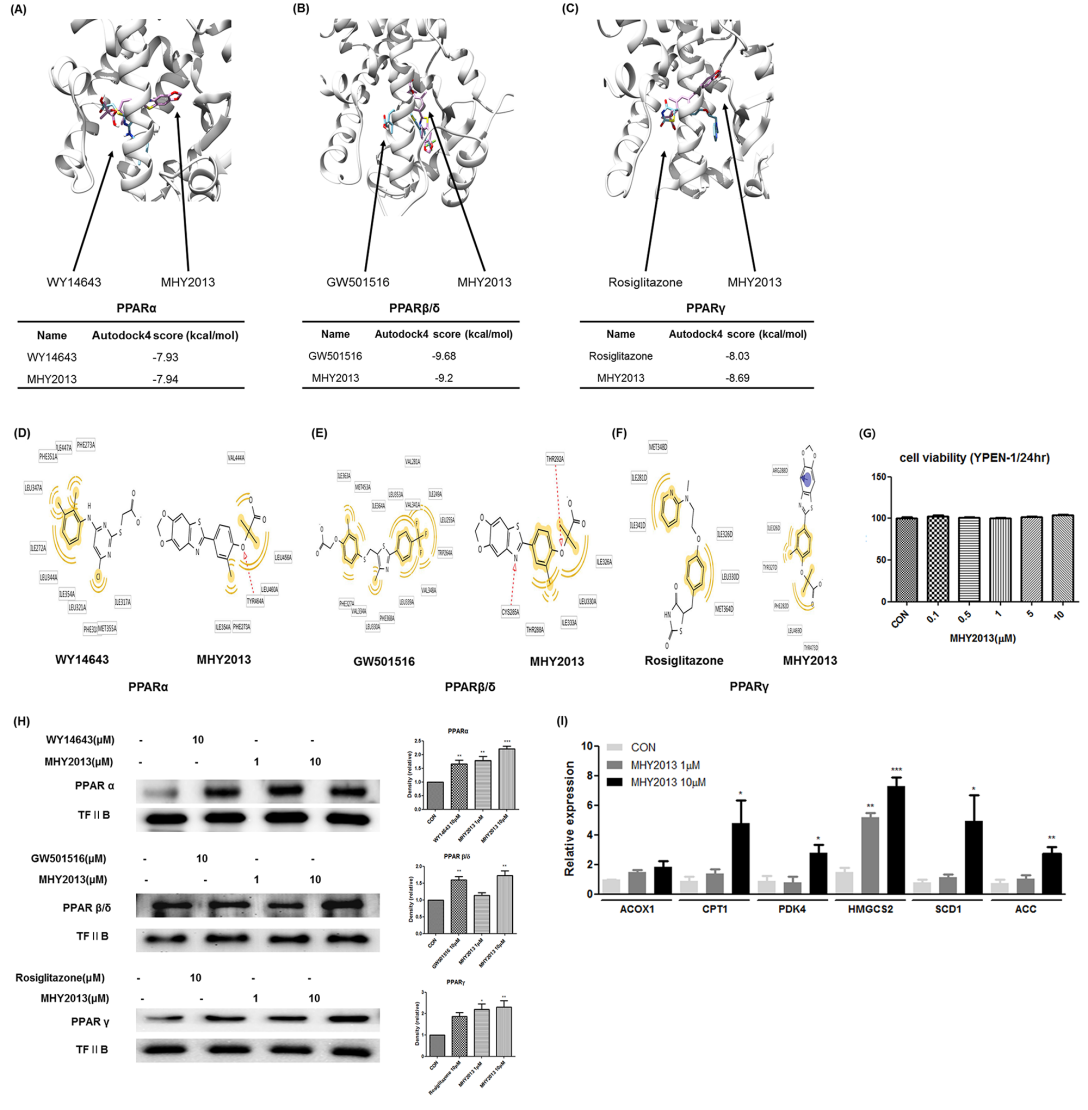
MHY2013 can directly bind to the three PPAR subtypes and increases their transcriptional activity **A, B, C**. Computational structure prediction for docking simulation between each of the three PPAR subtypes and MHY2013. The gray zone indicates the active site of PPAR subtypes (PDB ID: PPARα, 1K7L; PPARβ/δ, 1GWX; and PPARγ, 3DZY), magenta indicates MHY2013 and cyan indicates the respective positive control for each of the three PPAR subtypes. The small tables indicate the predicted docking score of MHY2013 and respective positive control with three PPAR subtypes. **D, E, F**. The binding sites of MHY2013 and the respective positive control for each of the three PPAR subtype are shown. The yellow indicates the hydrophobic interaction, purple indicates the aromatic interaction, and red indicates the hydrogen bond. **G**. YPEN-1 cells were treated with various concentrations (0.1, 0.5, 1, 5 and 10μM) of MHY2013 for 24 h, and then cell viability assay was performed (n = 4). **H**. The nuclear levels of PPARs in MHY2013-treated cells for 90 min were analyzed by western blotting, and the blots were quantified by densitometry (n = 3). Transcription factor II B (TF II B) was used as the loading control. **I**. Levels of PPAR target genes (PPARα: *ACOX1* and *CPT1*, PPARβ/δ: *PDK4* and *HMGCS2*, PPARγ: *SCD1* and *ACC*) in MHY2013-treated YPEN-1 cells for 5 h were analyzed by quantitative reverse transcription–polymerase chain reaction (qRT–PCR). The data are shown as the mean ± SEM (n = 3). *, *p* < 0.05 vs. CON; **, *p* < 0.01 vs. CON; ***, *p* < 0.001 vs. CON.

To investigate whether MHY2013 can bind to the three PPAR subtypes, docking simulation was conducted using the AutoDock 4.2 program. The structure images of the three PPAR subtypes from the docking simulation showed that MHY2013 might directly bind to the three PPAR subtypes (Figure 2A, B, C). To predict the binding energy, the docking topologies of WY14643, GW501516, rosiglitazone, and MHY2013 were simulated to the co-crystal structure of each PPAR subtype. The binding affinities of WY14643, GW501516, and rosiglitazone to PPARα, PPARβ/δ, and PPARγ, respectively, were −7.93, −9.68, and −8.03 kcal/mol, respectively, whereas the binding affinities of MHY2013 to PPARα, PPARβ/δ, and PPARγ were −7.94, −9.2, and −8.69 kcal/mol, respectively (Figure 2A, B, C). These results suggest that the binding affinity of MHY2013 to each PPAR subtype may be comparable to that of the positive controls. To predict the binding residues of the three PPAR subtypes, which correlated for each positive control and MHY2013, the LigandScout 3.1 program was used. MHY2013 might form a hydrogen bond with the TYR464A residue and hydrophobic bonds with the ILE354A, PHE273A, LEU460A, LEU456A, and VAL444A residues of PPARα (Figure 2D). In the case of PPAR β/δ, MHY2013 might form hydrogen bonds with the CYS285A and THR292A residues and hydrophobic bonds with the THR268A, ILE333A, LEU330A, and ILE326 residues (Figure 2E). MHY2013 might interact with PPARγ by forming an aromatic bond with the ARG288D residue and hydrophobic bonds with the ILE326D, TYR327D, PHE282D, LEU469D, and TYR473D residues (Figure 2F). In particular, MHY2013 formed hydrophobic bonds with the ILE354A residue of PPARα, LEU330A residue of PPARβ/δ, and ILE326D residue of PPARγ, in common with each PPAR subtype agonist (Figure 2D, E, F). These data indicate that MHY2013 may directly bind to all PPAR subtypes with affinities comparable to those of well-known PPAR subtype agonists.

### MHY2013 increases transcriptional activity of three PPAR subtypes

To investigate whether MHY2013 has cytotoxicity that was reported in some well-known PPAR agonists, including fenofibrate and pioglitazone, we carried out 3-(4,5-dimethylthiazol-2-yl)-2,5-diphenyltetrazolium bromide (MTT) assays and proved that MHY2013 had no cytotoxicity (up to 10 μM) in YEPN-1 cells during 24h incubation (Figure 2G). Because PPARs activated by ligand binding are translocated to the nucleus, we investigated whether MHY2013 induced translocation of PPARs to the nucleus. Immunofluorescence staining revealed that the nuclear distribution of the three PPAR subtypes were increased in the MHY2013-treated groups compared with those treated with the vehicle (DMSO) and each PPAR positive control (Supplementary Figure 2). Furthermore, western blotting results showed that the nuclear protein levels of the three PPAR subtypes were increased dose-dependently by the MHY2013 treatment (Figure 2H). Consistently, the mRNA levels of the target genes of PPARα (*ACOX1* and *CPT1*), PPARβ/δ (*PDK4* and *HMGCS2*), and PPARγ (*SCD1* and *ACC*) were significantly elevated by the MHY2013 treatment (Figure 2I). These results confirm that MHY2013 is a strong PPAR pan agonist targeting all PPAR subtypes.

### Effects of MHY2013 on blood profile and insulin sensitivity in obesity model

**Figure 3:**
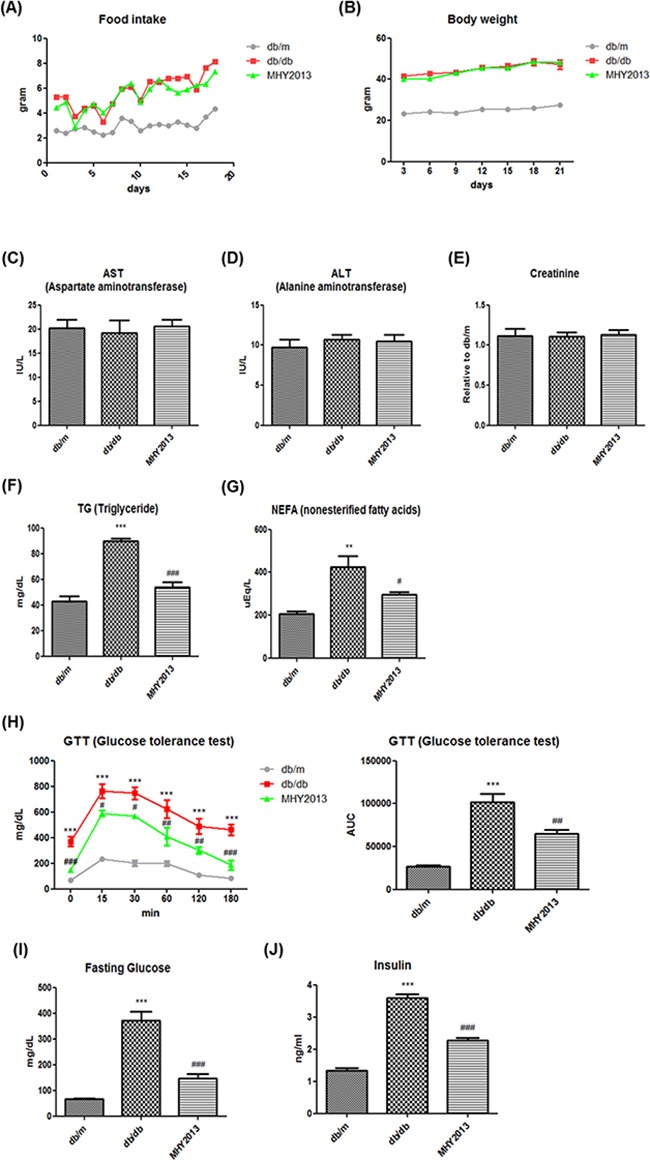
MHY2013 exhibits potent glucose- and lipid-lowering effects without changing the body weight in genetically obese db/db mice The mice were orally treated with the vehicle (water) or 5 mg/kg/day of MHY2013 for three weeks (n = 5). **A**. Food intake. **B**. Body weight. **C**. Serum aspartate aminotransferase (AST), **D**. alanine aminotransferase (ALT), **E**. creatinine, **F**. triglyceride (TG), and **G**. non-esterified fatty acid (NEFA) levels. **H**. Glucose concentrations were determined using an intraperitoneal glucose tolerance test (ipGTT) [inset: area under the curve (AUC), mg/dL × min] by administration of 1.5 g/kg glucose after overnight fasting. **I**. Fasting serum glucose and **J**. insulin levels. The data are shown as the mean ± SEM (n = 5). *, *p* < 0.05 vs. db/m; **, *p* < 0.01 vs. db/m; ***, *p* < 0.001 vs. db/m; #, *p* < 0.05 vs. db/db; ##, *p* < 0.01 vs. db/db; ###, *p* < 0.001 vs. db/db.

Obesity is generally accompanied by severe alterations in blood nutrient and metabolite profiles, which are closely related to metabolic syndrome, including insulin resistance and dyslipidemia. To evaluate the beneficial effects of MHY2013 on the blood profile, genetically obese db/db mice were administered with MHY2013 (5 mg/kg/day) by oral gavage for three weeks. There were no differences in the food intake and body weight between the groups (Figure 3A, B). We first tested whether MHY2013 had toxic effects *in vivo*. Serum aspartate transaminase (AST) and alanine transaminase (ALT) level used to detect the liver injury and the serum creatinine level used to detect the renal injury were unaffected by the MHY2013 treatment (Figure 3C, D, E), suggesting that MHY2013 has no severe toxicity for the liver and kidney. Then, we examined its effect on the blood profile associated with glucose and lipid metabolism. As expected, triglyceride (TG), non-esterified fatty acid (NEFA), fasting glucose, and circulating insulin levels highly increased in the db/db mice compared with the db/m mice, confirming the development of dyslipidemia and insulin resistance in the db/db mice (Figure 3F, G, I, J). However, MHY2013 reduced the serum levels of TGs and NEFAs in the db/db mice (Figure 3F, G). The GTT showed that MHY2013 improved the obesity-induced glucose intolerance (Figure 3H). Moreover, the MHY2013 treatment significantly reduced the fasting blood glucose and serum insulin levels in the db/db mice, without affecting the body weight (Figure 3I, J). These results suggest that MHY2013 improves obesity-induced dyslipidemia and insulin resistance, and the improvement is not a secondary effect of the body weight reduction.

### Effects of MHY2013 on liver of obese mice

**Figure 4:**
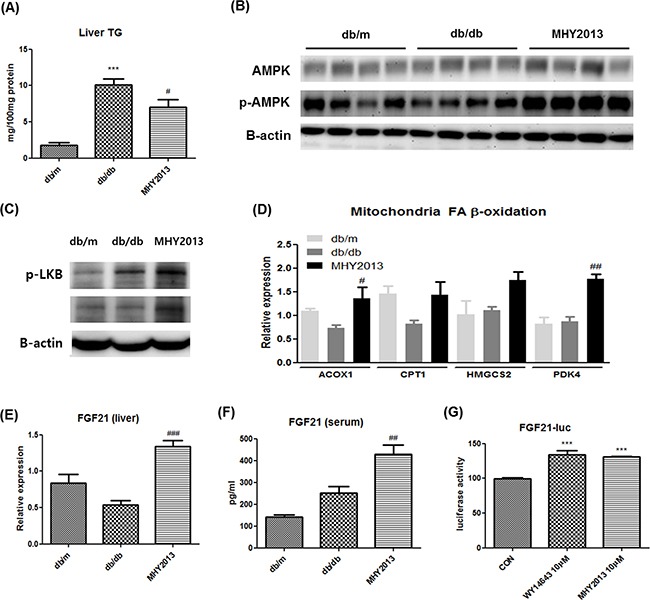
MHY2013 ameliorates hepatic steatosis and increases serum FGF21 levels The mice were orally treated with vehicle (water) or 5mg/kg/day of MHY2013 for three weeks (n = 5). **A**. Hepatic triglyceride (TG) contents. **B**. Activated p-AMPK (Thr172), total AMPK and **C**. p-LKB1 (Ser431) were analyzed in liver tissue by western blotting. β-Actin was used as the loading control. **D**. mRNA levels of fatty acid oxidation-related genes (*ACOX1*, *CPT1*, *HMGCS2*, and *PDK4*) were analyzed in liver tissue by qRT–PCR. **E**. mRNA levels of fibroblast growth factor 21 (FGF21) in liver tissue were analyzed by qRT–PCR. **F**. Serum FGF21 levels were examined using the Mouse/Rat FGF-21 Quantikine ELISA Kit. The data are shown as the mean ± SEM (n = 5). *, *p* < 0.05 vs. db/m; **, *p* < 0.01 vs. db/m; ***, *p* < 0.001 vs. db/m; #, *p* < 0.05 vs. db/db; ##, *p* < 0.01 vs. db/db; ###, *p* < 0.001 vs. db/db. **G**. The FGF21–LUC plasmid was transfected into HepG2 cells. Twenty-four hours after the transfection, cells were treated with the indicated compound for 5 h. The data are shown as the mean ± SEM (n = 5) luciferase activity, considered to represent the PPAR transcriptional activity. *, *p* < 0.05 vs. CON; **, *p* < 0.01 vs. CON; ***, *p* < 0.001 vs. CON.

Hepatic steatosis is commonly found together with insulin resistance in obesity. We examined the beneficial effect of MHY2013 on the liver and found that the compound ameliorated the obesity-induced hepatic steatosis as evidenced by a significant decrease in the hepatic TG concentration (Figure 4A). As a potential factor underlying the MHY2013-mediated decrease in the hepatic TG level, we investigated AMP-activated protein kinase (AMPK)/β-oxidation signaling because diverse studies have indicated that all PPAR subtypes induce AMPK activation through various mechanisms, such as a physical interaction, phosphorylation, and upregulation of AMPK activation-related proteins [9]. Phosphorylation of AMPKα1/2 (Thr172) was significantly increased in the liver but not adipose tissue and muscle of the MHY2013-treated db/db mice compared to those of the vehicle-treated db/db mice (Figure 4B and Supplementary Figure 3A, B). Moreover, MHY2013 induced phosphorylation of LKB1 (Ser431) known as upstream of AMPK in the liver (Figure 4C). Consistently, the mRNA expression levels of fatty acid oxidation-related genes, including *ACOX1*, *CPT1*, *HMGCS2*, and *PDK4*, were increased in the livers of the MHY2013-treated db/db mice (Figure 4D), indicating that the increased AMPK/β-oxidation signaling contributes to the improvement of hepatic steatosis.

FGF21 is a recently described hepatokine, primarily regulated by PPARα. Numerous studies have revealed the pharmaceutical importance of FGF21 for treating metabolic syndrome, which is due to its strong insulin-sensitizing and thermogenic effects on adipose tissues [10]. Because it is possible that MHY2013 activates PPARα via direct binding, we examined whether the compound stimulated the FGF21 production in the liver. The data showed that the mRNA expression levels of FGF21 were markedly increased in the livers of the db/db mice by the MHY2013 treatment (Figure 4E). Consistently, the serum level of FGF21 was also elevated by MHY2013 (Figure 4F). The FGF21–luciferase assay using HepG2 cells further revealed that MHY2013 highly increased the FGF21–luciferase activity to the level comparable to that of the PPARα agonist WY14643-treated group (Figure 4G). These data indicate that the MHY2013-mediated activation of PPARα transcriptionally upregulates FGF21 in the liver, contributing to the FGF21 increase in blood.

### Effects of MHY2013 on adipose tissue of obese mice

Although their expression levels are different, all PPAR subtypes have been shown to affect adipocyte metabolism. Indeed, a strong thermogenic effect of FGF21 on adipose tissues has been well established. To reveal the net effects of MHY2013 on white adipose tissue (WAT), we examined various signaling pathways related to fatty acid oxidation, thermogenesis, and inflammation.

**Figure 5:**
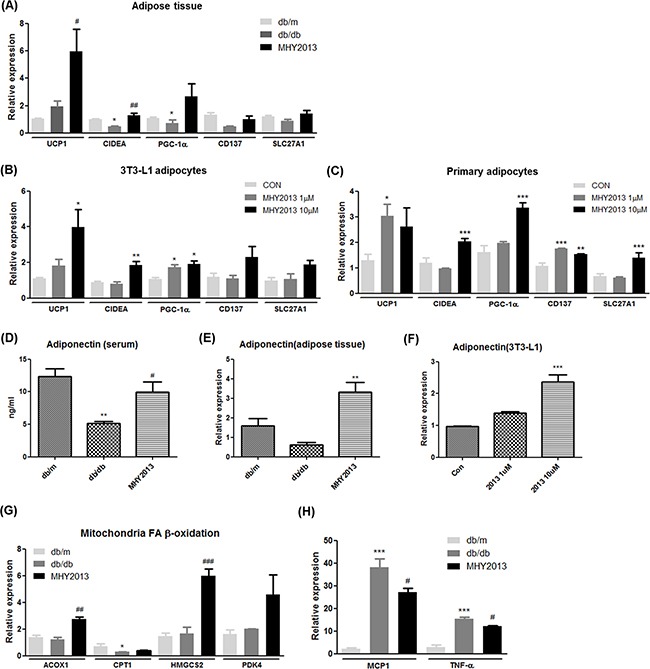
MHY2013 promotes adipose tissue browning and increases adiponectin levels The mice were orally treated with the vehicle (water) or 5 mg/kg/day of MHY2013 for three weeks (n = 5). **A**. In adipose tissue **B**. 3T3-L1 adipocytes and **C**. primary adipocyte from subcutaneous fat, mRNA expression of browning markers (*UCP1*, *CIDEA*, *PGC-1α*, *CD137*, and *SLC27A1*) was examined by qRT–PCR. **D**. In adipose tissue, mRNA expression of adiponectin was evaluated by qRT–PCR. **E**. Serum adiponectin levels were analyzed using the Mouse Adiponectin ELISA Kit. **F**. In 3T3-L1 cells, mRNA expression of adiponectin was evaluated by qRT–PCR. In adipose tissue, the mRNA levels of **G**. fatty acid oxidation-related genes (*ACOX1*, *CPT1*, *HMGCS2*, and *PDK4*) and **H**. inflammatory cytokines (*MCP1* and *TNF-α*) were analyzed by qRT–PCR. For mouse studies, *, *p* < 0.05 vs. db/m; **, *p* < 0.01 vs. db/m; ***, *p* < 0.001 vs. db/m; #, *p* < 0.05 vs. db/db; ##, *p* < 0.01 vs. db/db; ###, *p* < 0.001 vs. db/db. For cell experiments, *, *p* < 0.05 vs. CON; **, *p* < 0.01 vs. CON; ***, *p* < 0.001 vs. CON.

The mRNA expression levels of browning markers such as *UCP1* and *CIDEA* were significantly increased in adipose tissue of the MHY2013-treated db/db mice compared with the vehicle-treated db/db mice (Figure 5A). Other browning markers, such as *PGC1-α*, *CD137*, and *SLC27A1*, also showed a tendency to be slightly increased by MHY2013 (Figure 5A). To check whether MHY2013 directly induces the browning of white adipocytes, 3T3-L1 pre-adipocytes and primary adipocytes isolated from subcutaneous fat of C57/BL6 mice were differentiated into adipocytes for seven days with or without MHY2013. As a result, MHY2013 dose-dependently increased the mRNA levels of browning markers such as *UCP1*, *CIDEA*, and *PGC1-α* in the 3T3-L1 and primary adipocytes (Figure 5B, C). To further examine the adipose browning effect of MHY2013, we observed the morphology of lipid droplets because brown adipocytes are characterized by a multi-locular lipid droplet structure. MHY2013 appeared to alter the morphology of adipocytes toward a multi-locular lipid droplet structure in 3T3-L1 cells (Supplementary Figure 4A). These data suggest that MHY2013 induces browning of WAT.

One of the important functions of adipose tissue is secretion of adipokines to regulate systemic nutrient metabolism. Adiponectin is probably one of the best-known adipokines, which elevates the insulin sensitivity and fatty acid oxidation and reduces the inflammation. Indeed, it has been shown that beneficial effects of PPAR subtype agonists are partially mediated via increasing adiponectin levels. Because adiponectin gene expression in adipocytes is induced by all three PPAR subtypes, we evaluated adiponectin levels in the MHY2013-treated db/db mice. MHY2013 notably increased the mRNA expression level of adiponectin in adipose tissue (Figure 5D). Consistently, the blood adiponectin level was higher in the MHY2013-treated than in the vehicle-treated db/db mice (Figure 5E). To investigate whether MHY2013 directly regulates the level of adiponectin in adipocytes, we measured the adiponectin mRNA expression level in MHY2013-treated 3T3-L1 adipocytes. The mRNA expression of adiponectin was greatly increased by MHY2013 in the 3T3-L1 cells (Figure 5F). These data demonstrate that the MHY2013-mediated increase in adiponectin levels contributes to the improvement of obesity-related insulin resistance and dyslipidemia.

To further examine the effects of MHY2013 on adipose tissue, we determined the mRNA expression levels of genes associated with fatty acid oxidation and inflammation signaling pathways. The mRNA expression levels of fatty acid oxidation-related genes (*ACOX1*,*CPT1*, *HMGCS2*, and *PDK4)* were increased in adipose tissue of the MHY2013-treated db/db mice and 3T3-L1 cells (Figure 5G and Supplementary Figure 4B). MHY2013 also reduced the mRNA expression levels of inflammatory genes such as *MCP1* and *TNF-α* (Figure 5H). Although we showed that the MHY2013 treatment increased the FGF21 expression in the liver, no significant change in FGF21 expression was observed in adipose tissue (Supplementary Figure 4C). Together, these data indicate that MHY2013 not only stimulates the metabolic pathways for energy expenditure but also inhibits inflammatory signaling, thereby contributing to maintaining metabolically healthy adipose tissue.

### Effects of MHY2013 on skeletal muscle of obese mice

**Figure 6:**
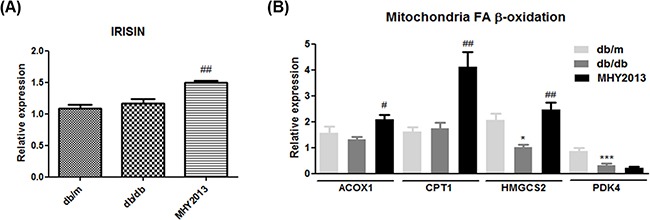
MHY2013 increased mRNA expression of irisin and fatty acid oxidation-related genes The mice were orally treated with the vehicle (water) or 5 mg/kg/day of MHY2013 for three weeks (n = 5). In muscle, mRNA levels of **A**. IRSIN and **B**. fatty acid oxidation-related genes (*ACOX1*, *CPT1*, *HMGCS2*, and *PDK4*) were analyzed by qRT–PCR. *, *p* < 0.05 vs. db/m; **, *p* < 0.01 vs. db/m; ***, *p* < 0.001 vs. db/m; #, *p* < 0.05 vs. db/db; ##, *p* < 0.01 vs. db/db; ###, *p* < 0.001 vs. db/db.

To investigate whether MHY2013 has beneficial effects on skeletal muscle, we measured mRNA expression levels of fatty acid oxidation-related genes and irisin, a recently identified myokine that improves obesity-related metabolic syndrome. MHY2013 increased mRNA expression levels of ACOX1, CPT1, HMGCS2, and irisin in the skeletal muscle (Figure 6A and 6B). Although further studies are necessary for proving beneficial effects of MHY2013 on skeletal muscle, these data shows that MHY2013 may increase fatty acid oxidation and irisin production in skeletal muscle.

## DISCUSSION

Through *in silico* docking simulation and multiple biological analysis, we screened MHY2013 as the strongest agonist for all PPAR subtypes among six 2-methyl-2-(*o*-tolyloxy)propanoic acid derivatives. When injected orally in db/db mice, MHY2013 greatly improved obesity-induced insulin resistance, dyslipidemia, and hepatic steatosis without a change in body weight and apparent signs of hepatic or renal toxicity. Although the underlying mechanisms should be diverse considering numerous effects of PPARs in multiple tissues, the elevation of beneficial hormones including FGF21 and adiponectin, WAT browning signaling, and fatty acid oxidation signaling in liver and skeletal muscle may synergistically contribute to the beneficial effects of MHY2013. Thus, MHY2013 may be a novel pharmaceutical agent that can be used for intervention in obesity-induced metabolic syndrome.

Previous studies have revealed that there are major drawbacks in selective PPAR agonists, such as a body weight gain and dyslipidemia. As an alternative, drugs targeting multiple PPARs have been developed. Although bezafibrate was first clinically tested as a PPAR pan agonist to improve metabolic disorders [11, 12], they caused some mooted points like severe hepatic and renal toxicity and low potency [13, 14]. Our data showed that MHY2013 did not accompany the known side effects including body weight gain and hepatic and renal toxicity. When compared to a PPAR α/γ dual agonist MHY908 [15, 16], MHY2013 appears to be a stronger activator for PPARs than MHY908 (unpublished data). We assume that the balanced activation of three PPAR subtypes by MHY2013 may contribute to the beneficial effects on metabolic syndrome with minimizing known side effects by other PPAR agonists.

The PPAR subtypes regulate the expression of endocrine hormones that can influence glucose and lipid metabolism [17]. FGF21 is a hormone secreted from the liver and adipose tissues in response to activation of PPARα and PPARγ respectively. FGF21 has been considered a promising intervention target for metabolic diseases, including fatty liver, obesity, and diabetes due to its insulin-sensitizing and thermogenesis-inducing effects [18]. Our data showed that FGF21 was increased in the liver and serum of the MHY2013-treated db/db mice, but the mRNA expression of FGF21 did not change in the the WAT. Therefore, we assume that the liver is the main contributor to the increase in serum FGF21 level by MHY2013 treatment. Besides FGF21, a well-known insulin-sensitizing hormone adiponectin is also increased in the serum of the MHY2013-treated mice possibly due to the upregulation of adiponectin expression in WAT. Considering MHY2013 elevated the mRNA expression of adiponectin in 3T3-L1 adipocyte cells, the compound may directly upregulate adiponectin expression in adipocytes by activating PPARγ that are well-known regulators of adiponectin transcription [19].

Moreover, MHY2013 induced AMPK activation in the liver. Because AMPK activation has been shown to stimulate the energy expenditure, including fatty acid oxidation, the MHY2013-mediated increase in fatty acid oxidation signaling is likely associated with the AMPK activation in the liver. Furthermore, it is very likely that the utilization of fatty acids in the liver may contribute to the reduced TG level in circulation in the MHY2013-treated mice because it has been reported that the increased fatty acid oxidation in the liver lowers serum lipid levels [20].

Adipose tissue browning is important for ameliorating obesity-related metabolic diseases because it prevents the excessive energy storage and inflammation by dissipating energy through heat [21]. MHY2013 increased the fatty acid oxidation and browning-related gene expression and decreased the inflammatory gene expression in WAT of the obese mice. Thus, it is likely that the fatty acid oxidation-derived energy may be dissipated through heat generation as a result of MHY2013 treatment. Furthermore, the increase in fatty acid oxidation and thermogenic signaling in WAT may contribute to the notable decrease in circulating NEFA levels in the MHY2013-treated db/db mice. However, the mechanisms underlying MHY2013-mediated increase in WAT browning signaling are not directly examined in the current study. Considering other studies showing that all PPAR subtypes stimulate WAT browning with hormone-dependent and -independent manners [21–24], we assume that the MHY2013-mediated increases in the expression of WAT browning markers and the formation of multi-locular lipid droplets in WAT are probably due to the MHY2013 agonism for all PPAR subtypes in multiple tissues and the PPAR agonism-dependent secretion of thermogenic activators, including FGF21 and adiponectin.

Although thermogenic signaling was increased in WAT, the body weight was unaltered in the mice treated with MHY2013. We assume that the WAT browning effect of MHY2013 may not be strong enough to reduce body weight or that other effects of all three PPAR subtype agonism by MHY2013 may offset the WAT browning-mediated body weight regulation. For instance, FGF21 has been shown to decrease body weight and improve metabolic profile in obese mice [25, 26]. On the other hand, adiponectin ameliorates metabolic profile although it increases body weight evidenced by clearly increased insulin sensitivity, healthier blood lipid profile, and less lipid accumulation in liver despite highly increased body weight in adiponectin-overexpressed ob/ob mice [27]. Thus, the net effect of MHY2013 may be the amelioration of metabolic profile without decreasing body weight.

In conclusion, the balanced activation of the three PPAR subtypes by MHY2013 improved obesity-induced insulin resistance, hepatic steatosis, and dyslipidemia without weight gain and severe toxicity. Although all the metabolic signaling pathways and target tissues are not examined in the current study, we assume that the elevation of beneficial hormones including FGF21 and adiponectin, WAT browning signaling, and fatty acid oxidation signaling in liver and skeletal muscle may synergistically contribute to the beneficial effects of MHY2013.

Our present study supports the concept of a pan PPAR therapeutic approach to conditions which comprise the metabolic syndrome. Further studies are needed to enable MHY2013 clinical use for targeting various metabolic diseases.

## MATERIALS AND METHODS

### Animal experiments

Male, 8-week-old C57BLKS/J-lean (db/m) and C57BLKS/J-db/db mice were purchased from Japan SLC and acclimated to the animal care facility for seven days before the experiments. Animals were housed in an air-conditioned atmosphere under a 12 h light/dark cycle and were given free access to a standard rodent chow (Samtako) and water. The animal study was approved by the Institutional Animal Care Committee of Pusan National University (2, Busandaehak-ro 63beon-gil, Geumjeong-gu, Busan, Republic of Korea) and performed in accordance with the guidelines for animal experiments issued by Pusan National University. Vehicle and MHY2013 (5 mg/kg/day) were injected by oral gavage for three weeks. The mice were monitored daily for food intake and once in three days for body weight. After two weeks, a glucose tolerance test (GTT) was performed by intraperitoneal injection of 1.5 g/kg glucose after overnight fasting. Blood was taken from the tail at indicated time points, and the glucose concentration was determined with a glucometer (Accu-Chek, Roche Diagnostics). The animals were euthanized after three weeks, and serum and tissues were collected.

### *In Silico* protein–ligand docking simulation

The crystal structures of human PPARα, PPARβ/δ, and PPARγ were obtained from the Protein Data Bank (PDB ID: PPARα, 1K7L; PPARβ/δ, 1GWX; and PPARγ, 3DZY) and used as the targets in docking calculations. We used the AutoDock 4.2 program and the tool's manual because of its automated docking capabilities. To define the docking pockets of the three PPAR subtypes, we used a set of predefined active sites of human PPARs. Docking simulations were performed between the three PPAR subtypes and MHY2013. To prepare compounds for the docking simulation, we performed the following steps: [1] 2D structures were converted into 3D structures, [2] charges were calculated, and [3] hydrogen atoms were added using the ChemOffice program (http://www.cambridgesoft.com). In addition, the LigandScout 3.0 program was used to generate a pharmacophore model and to predict possible hydrogen-bonding residues between the three PPAR subtypes and MHY2013. AMBER ff99SB force-field parameter was applied for calculating ligand molecules. The docking protocol was validated by docking co-crystallized ligand structure. Energy evaluations were 2500000 and population size was 150.

### Cell culture system and adipocytes differentiation

YPEN-1, a rat prostate endothelial cell line, and HepG2, a human liver cancer cell line, were purchased from the American Type Culture Collection (Manassas, VA, USA). 3T3-L1, a murine pre-adipocyte cell line, was obtained from Dr. Hyeung-Rak Kim (Pukyoung National University, Pusan, Korea). Cells were maintained in Dulbecco's modified Eagle's medium containing 5% (YPEN-1) or 10% (HepG2 and 3T3-L1) fetal bovine serum (Gibco, Grand Island, NY, USA), 100 U/mL penicillin, and 100 μg/mL streptomycin (all from Hyclone, Inc., Logan, UT, USA) at 37°C in a 5% CO_2_ atmosphere. For adipocytes differentiation, cells were cultured to confluence (day 0) and then exposed to the differentiation mixture (0.5 mm 3-isobutyl-1-methylxanthine, 1 μM dexamethasone, and 10 μg/mL insulin). After 48 h of incubation, the cells were maintained in a medium containing 10 μg/mL insulin until harvest on day 7.

### Primary adipocytes culture

Subcutaneous fat pads from 8-week-old mice were excised, minced and digested in HEPES buffer with type1 collagenase at 37°C for 90 min. The cells were diluted in 1% FBS-DMEM buffer. Diluted cell suspension was filtered with 70 μm nylon mesh to remove undigested tissues and then centrifuged at 500 g for 15 min to sediment clumps. The supernatant was removed and red blood cell (RBC) lysis buffer was added and gently suspended. After 5 min incubation at room temperature, equal volume of 10% FBS-DMEM was added and then filtered with 40 μm nylon mesh to remove endothelial cell clumps. Filtered cell suspension was centrifuged at 500 g for 5min and all but 2-3ml of the supernatant was removed. The pellet was suspended with 10% FBS-DMEM. The cell suspension was counted and seeded.

### Cell viability assay

The cell viability assay was carried out using 3-(4,5-dimethylthiazol-2-yl)-2,5-diphenyltetrazolium bromide (MTT; DoGen, Korea). YPEN-1 cells (1 × 10^4^) were plated in each well of a 96-well cell culture plate and allowed to attach overnight. After the cells were exposed to MHY2013 at concentrations ranging from 1 to 10 μM for 24 h, the MTT reagent was added to each well, and the plate was incubated for 1 h. Absorbance in each well was determined at 560 nm using a microplate reader

### Luciferase assay

For a peroxisome proliferator response element (PPRE)-driven luciferase assay, 5 × 10^4^ YPEN-1 cells were seeded per well into a 48-well cell culture plate. The PPRE-X3-TK-LUC plasmid (0.5 μg) (a kind gift from Dr. Christoper K. Glass, University of California, San Diego, CA, USA) and 0.05 μg of the PPARα, PPARβ/δ, and PPARγ expression vectors (kind gifts from Dr. Han Geuk Seo, Konkuk University, Seoul, South Korea) were transfected into the cells using 1 μL of the Lipofectamine 2000 reagent (Invitrogen, Carlsbad, CA, USA) according to the manufacturer's instructions. For the FGF21–luciferase assay, 2 × 10^4^ HepG2 cells were seeded per well into a 96-well cell culture plate. The FGF21–LUC plasmid (0.1 μg) (a kind gift from Dr. Kook Hwan Kim, Yonsei University, Seoul, South Korea) was transfected into the cells using 0.1 μL of the Lipofectamine 3000 reagent (Invitrogen) according to the manufacturer's instructions. After the incubation for 24 h, the cells were treated with the vehicle[dimethyl sulfoxide (DMSO)], 10 μM MHY2013, WY14643 (a known PPARα agonist), rosiglitazone (a known PPARγ agonist), and GW501516 (a known PPARβ/δ agonist) for 5 h. Luciferase activity was measured using the One-Glo Luciferase Assay System (Promega, Madison, WI, USA) and a luminometer (Berthold Technologies GmbH & Co., Bad Wildbad, Germany).

### Immunocytochemistry

YEPN-1 cells (2 × 10^5^) were seeded in a 35-mm cover-glass-bottom dish and allowed to attach overnight. The cells were treated with 10 μM MHY2013, WY14643, rosiglitazone, and GW501516 for 1.5 h and washed twice with phosphate-buffered saline (PBS). Then, the cells were fixed in 4% paraformaldehyde for 30 min at room temperature and washed using PBS with 0.5% Triton X-100. After washing, the cells were blocked in ABS/0.1% Triton X-100/3% goat serum (ABS-TS) for 30 min. Subsequently, the cells were immunostained with rabbit anti-PPARα, anti-PPARβ/δ, and anti-PPARγ primary antibodies (all from Santa Cruz Biotechnology, Santa Cruz, CA, USA), diluted in ABS-TS (1:1,000), at 4°C overnight. The cells were washed three times with ABS-TS, then incubated with a secondary anti-rabbit IgG antibody labeled with Alexa Fluor-488 (Invitrogen) for 30 min, and washed with ABS. Cell nuclei were visualized by immunostaining with Hoechst 33342 (Invitrogen), and immunostained images of the three PPAR subtypes were acquired by confocal laser scanning microscopy (TCS SP2, Leica, Wetzler, Germany).

### Protein extraction and immunoblot analysis

For extraction of cytosol and nuclear fractions from cells and tissues, a cell pellet or tissue was suspended and homogenized in 10 mM Tris, pH 8.0, with 1.5 mM MgCl_2_, 1 mM dithiothreitol, 0.1% NP-40, and protease inhibitors, incubated on ice for 15 min, and then centrifuged at 12,000 rpm at 4°C for 10 min. The supernatants were used as the cytosolic fractions. The pellets were washed three times and re-suspended in 10 mM Tris, pH 8.0, with 50 mM KCl, 100 mM NaCl, and protease inhibitors, incubated on ice for 30 min, and then centrifuged at 12,000 rpm at 4°C for 10 min. The resultant supernatants were used as the nuclear fractions. The protein concentration was measured using the BCA assay (Thermo Scientific, Waltham, MA, USA). Samples were prepared in a gel buffer (12.5 mM Tris, 4% sodium dodecyl sulfate, 20% glycerol, 10% 2-mercaptoethanol, and 0.2% bromophenol blue, pH 6.8) and boiled for 5 min. Western blot assays were performed as previously described, with minor modifications [28]. Primary antibodies raised against PPARα, PPARβ/δ, PPARγ, AMPKα1/2, phospho-AMPKα1/2 (Thr172), phospho-LKB1 (Ser431), transcription factor II B (TFIIB), and β-Actin (all from Santa Cruz Biotechnology) were used.

### Isolation of RNA and quantitative real-time polymerase chain reaction

Tissue and cell RNA was purified using the TRIzol reagent (Invitrogen) according to the manufacturer's instruction. Total RNA (2.0 μg) treated with RNase-free DNase was reverse-transcribed with a cDNA synthesis kit from GenDEPOT (Barker, TX, USA). Quantitative polymerase chain reaction (qPCR) was performed using the THUNDERBIRD SYBR qPCR mix (TOYOBO Co., Osaka, Japan) and a CFX Connect System (Bio-Rad Laboratories, Inc., Hercules, CA, USA). The primer sequences are shown in Supplementary Table 2.

### Serum biochemical analysis and cytokine measurements

Serum glucose, cholesterol, triglycerides (TGs), non-esterified fatty acids (NEFAs), and creatinine were analyzed using kits from Bioassay Systems (Hayward, CA, USA). To measure the serum adiponectin level, the Mouse Adiponectin ELISA Kit (CircuLex Co.) was used. The serum fibroblast growth factor 21 (FGF21) level was evaluated using the Mouse/Rat FGF-21 Quantikine ELISA Kit (R&D Systems). Serum alanine aminotransferase (ALT) and aspartate aminotransferase (AST) were measured using a kit from Stanbio (Boerne, TX, USA).

### Liver triglyceride measurements

Liver samples were homogenized in ice-cold PBS. TGs were extracted with methanol/chloroform (1:2), dried, and suspended in 5% bovine serum albumin. The TG level was evaluated using a kit from Bioassay Systems.

### Statistical analysis

All data are expressed as the mean ± standard error of the mean (SEM). Statistical significance of the differences between groups was determined by one-way analysis of variance, followed by a Dunnett's test. An associated probability (*p* value) of < 0.05 was considered significant.

## SUPPLEMENTARY MATERIALS FIGURES AND TABLES


