# Correction: Physiological characterization of a novel PPAR pan agonist, 2-(4-(5,6-methylenedioxybenzo[d]thiazol-2-yl)-2-methylphenoxy)-2-methylpropanoic acid (MHY2013)

**DOI:** 10.18632/oncotarget.18922

**Published:** 2017-07-03

**Authors:** Hye Jin An, Bonggi Lee, Dae Hyun Kim, Eun Kyeong Lee, Ki Wung Chung, Min Hi Park, Hyoung Oh Jeong, Sung Min Kim, Kyoung Mi Moon, Ye Ra Kim, Seong Jin Kim, Hwi Young Yun, Pusoon Chun, Byung Pal Yu, Hyung Ryong Moon, Hae Young Chung

**Present:** The author name ‘Sung Min Kim’ is spelled incorrectly.

**Correct:** The proper spelling is ‘Seong Min Kim’.

Original article: Oncotarget. 2017; 8:16912-16924. https://doi.org/10.18632/oncotarget.14818

