# Supplementary material for: Physiological characterization of a novel PPAR pan agonist, 2-(4-(5,6-methylenedioxybenzo[d]thiazol-2-yl)-2-methylphenoxy)-2-methylpropanoic acid (MHY2013)
Source: Oncotarget. 2017 Jan 25;8(10):16912–24. doi: 10.18632/oncotarget.14818 (PMC5370010; doi:10.18632/oncotarget.14818)
Supplement: Supplementary file 1 [file oncotarget-08-16912-s001.pdf]

Physiological characterization of a novel PPAR pan agonist, 2-(4-(5,6-methylenedioxybenzo[*d*]thiazol-2-yl)-2-methylphenoxy)-2-methylpropanoic acid (MHY2013)

SUPPLEMENTARY APPENDICES

SUPPLEMENTARY METHOD : DESIGN AND SYNTHESIS OF MHY2013

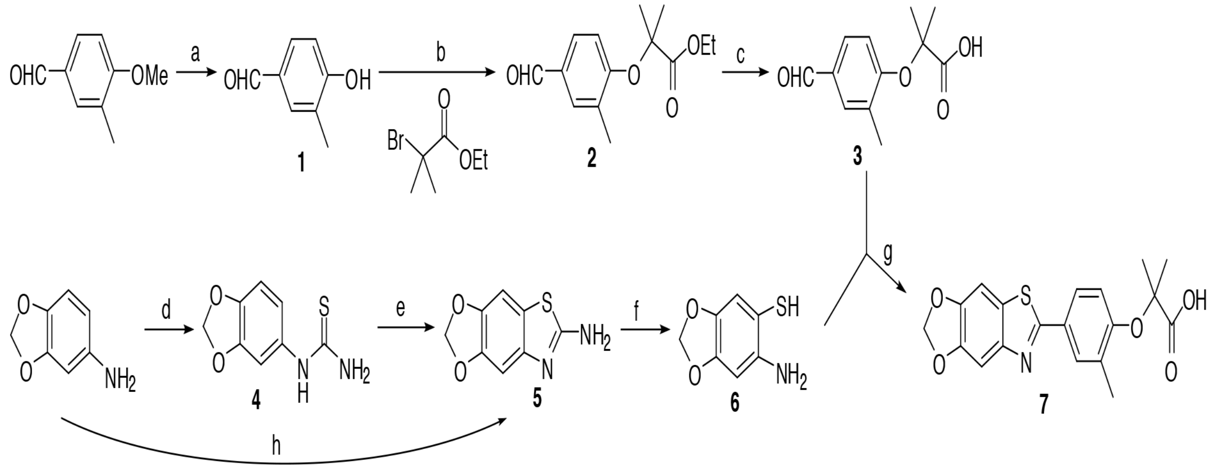

Reagents and conditions: (a) BBr_3, CH_2Cl_2, room temperature, 48 h; (b) 1 N sodium ethoxide, anhydrous ethanol (EtOH), dimethylformamide (DMF), reflux, 4 days; (c) 1 N NaOH, 1,4-dioxane, room temperature, 5 h; (d) i) NH_4SCN, benzoyl chloride, acetone, 50 °C, 1 h; ii) 2 N NaOH, reflux, 1 h; (e) i) Br_2, acetic acid, room temperature, 40 min; ii) 28% NH_4OH, room temperature, 1 h; (f) KOH, 2-methoxyethanol, H_2O, reflux, 4.5 h; (g) $Na_2S_2O_5$, DMF, 80 °C, 20 h; (h) KSCN, acetic acid, Br_2, room temperature, 22 h.

4-Hydroxy-3-methylbenzaldehyde (1)

To a stirred solution of 4-methoxy-3-methylbenzaldehyde (1.0 g, 6.66 mmol) in methylene chloride (5 mL) at 0 °C, boron tribromide (10 mL, 10.0 mmol, 1.0 M solution in methylene chloride) was added dropwise. The reaction mixture was stirred for 48 h at room temperature and concentrated in vacuum until the volume of the residue became 1.5 mL. The residue was treated with methanol (MeOH, 3.5 mL) at 0 °C, refluxed for 30 min, and then the volatiles were completely evaporated. Water (4 mL) was added to the residue, and the mixture was stirred at room temperature for 30 min and then extracted with diethyl ether. The organic layer was dried and concentrated under reduced pressure. The crude products were purified by silica gel column chromatography (methylene chloride/MeOH, 49:1) to give title compound **1** (98.6%) as a light orange-colored solid; melting point, 118.2–120.2 °C; ^{1}H NMR (500 MHz, DMSO-d_6) δ 10.52 (s, 1 H, O*H*), 9.74 (s, 1 H, C*H*O), 7.61 (d, 1 H, J = 1.5 Hz, 2-H), 7.58 (dd, 1 H, J = 1.5, 8.5 Hz, 5-H), 6.93 (d, 1 H, J = 8.5 Hz, 6-H), 2.16 (s, 3 H, 3-CH_3); ^{13}C NMR (100 MHz, DMSO-d_6) δ 191.7, 162.2, 132.9, 130.6, 128.9, 125.6, 115.5, 16.4.

Ethyl 2-(4-formyl-2-methylphenoxy)-2-methylpropanoate (2)

To a solution of 4-hydroxy-3-methybenzaldehyde **1** (878 mg, 6.45 mmol) and ethyl α-bromoisobutyrate (1.42 mL, 9.68 mmol) in anhydrous EtOH (3 mL) and DMF (7 mL), 1 N sodium ethoxide (9.67 mL, 9.67 mmol) was added dropwise. Every day, additional ethyl α-bromoisobutyrate (1.42 mL) and 1 N sodium ethoxide (9.67 mL) were added, and the reaction mixture was refluxed for four days. EtOH was removed under reduced pressure, and the residue was partitioned between diethyl ether and water. The organic layer was dried over $MgSO_4$, filtered, and concentrated under reduced pressure. The crude product was purified by silica gel column chromatography using hexane and ethyl acetate (13:1) as the eluent to give compound **2** (72.3%) as yellow oil; ^{1}H NMR (400 MHz, $CDCl_3$) δ 9.81 (s, 1 H, C*H*O), 7.67 (d, 1

H, *J* = 2.0 Hz, 3′-H), 7.56 (dd, 1 H, *J* = 2.0, 8.8 Hz, 5′-H), 6.65 (d, 1 H, *J* = 8.8 Hz, 6′-H), 4.20 (q, 2 H, *J* = 7.2 Hz, CH_2CH_3), 2.26 (s, 3 H, 2′-CH_3), 1.65 (s, 6 H, 3-H_3, 2-CH_3), 1.18 (t, 3 H, *J* = 7.2 Hz, CH_2CH_3); ^{13}C NMR (100 MHz, $CDCl_3$) δ 191.3, 173.9, 159.5, 132.2, 130.1, 129.8, 129.6, 114.6, 79.7, 61.9, 25.7, 16.9, 14.2; LRMS (ESI) *m/z* 251 $(M-H)^+$.

2-(4-Formyl-2-methylphenoxy)-2-methylpropanoic acid (3)

To a solution of compound **2** (240 mg, 0.96 mmol) in 1,4-dioxane (1 mL), 1 N NaOH aqueous solution (1.2 mL, 1.2 mmol) was added. After stirring for 5 h at room temperature, the reaction mixture was partitioned between methylene chloride and water. The aqueous layer was acidified with concentrated HCl. The resulting precipitate was extracted with methylene chloride, and the organic layer was dried over $MgSO_4$, filtered, and evaporated to give the title compound **3** (97.0%) as oil: ^{1}H NMR (400 MHz, $CDCl_3$) δ 9.81 (s, 1 H, CHO), 7.68 (d, 1 H, *J* = 2.0 Hz, 3′-H), 7.59 (dd, 1 H, *J* = 2.0, 8.4 Hz, 5′-H), 6.76 (d, 1 H, *J* = 8.4 Hz, 6′-H), 2.26 (s, 3 H, 2′-CH_3), 1.65 (s, 6 H, 3-H_3, 2-CH_3); ^{13}C NMR (100 MHz, $CDCl_3$) δ 191.8, 178.0, 159.3, 132.5, 130.2, 130.0, 129.8, 115.1, 79.4, 25.6, 16.9; LRMS (ESI) *m/z* 221 $(M-H)^-$.

1-(Benzo[*d*][1,3]dioxol-5-yl)thiourea (4)

To a solution of ammonium thiocyanate (3.2 g, 42.0 mmol) in acetone (30 mL), benzoyl chloride (3.9 mL, 33.6 mmol) was added dropwise. The reaction mixture was heated at 50 °C for 20 min, and then a solution of 3,4-(methylenedioxy)aniline (5.0 g, 36.5 mmol) in acetone (35 mL) was added dropwise. After the reaction mixture was stirred at 50 °C for 1 h, water was poured into the reaction mixture. The resulting precipitate was collected by filtration and washed with water. The obtained filter cake was refluxed with 2 N NaOH (60 mL) for 1 h, and after cooling, the reaction mixture was poured into water. The resulting precipitate was filtered and washed with water to give thiourea **4** (3.4 g, 47.5%) as a white solid; ^{1}H NMR (400 MHz, DMSO-d_6) δ 9.47 (s, 1 H), 7.25 (brs, 2 H), 6.94 (s, 1 H), 6.81 (d, 1 H, *J* = 8.4 Hz), 6.64 (d, 1 H, *J* = 8.4 Hz), 5.96 (s, 2 H); ^{13}C NMR (100 MHz, DMSO-d_6) δ 181.8, 147.8, 145.2, 133.5, 117.9, 108.6, 106.6, 101.9.

2-Amino-5,6-methylenedioxybenzo[*d*]thiazole (5)

To a suspension of compound **4** (1.71 g, 8.71 mmol) in acetic acid (30 mL), bromine (0.45 mL, 8.78 mmol) at 0 °C was added, and the reaction mixture was stirred for 40 min at room temperature. Diethyl ether was poured into the reaction mixture, and the resulting precipitate was collected by filtration. The obtained precipitate was suspended in 28% ammonium hydroxide solution (15 mL) and stirred for 1 h at room temperature. The remaining solid was filtered to give title product **5** (1.454 g, 85.9%) as a solid.

Another synthetic method: To a stirred solution of 3,4-(methylenedioxy)aniline (1.2 g, 8.75 mmol) in acetic acid (10 mL), a solution of potassium thiocyanate (3.4 g, 34.99 mmol) in acetic acid (18 mL) was added slowly, and the reaction mixture was stirred at room temperature for 30 min. After cooling to 0–5 °C, bromine (0.48 mL, 9.37 mmol) in acetic acid (10 mL) was added to the reaction mixture slowly, and the reaction mixture was stirred at room temperature for 22 h. After water was added, the precipitate generated was filtered and washed with water. To the obtained filter cake, water was added, and the suspension was neutralized with 28% ammonium hydroxide. The remaining solid was filtered and washed with water to give title product **5** (1.37 g, 80.6%) as a solid; ^{1}H NMR (500 MHz, DMSO-d_6) δ 7.23 (s, 1 H), 7.19 (s, 2 H, NH_2), 6.91 (s, 1 H), 5.92 (s, 2 H, CH_2); ^{13}C NMR (100MHz, DMSO-d_6) δ 166.7, 147.7, 146.9, 143.0, 122.6, 101.7, 101.4, 99.9.

6-aminobenzo[*d*][1,3]dioxole-5-thiol (6)

A stirred solution of compound **5** (1.0 g, 5.15 mmol) and potassium hydroxide (5.88 g, 104.79 mmol) in water (6 mL) and 2-methoxyethanol (6 mL) were refluxed for 4.5 h. After cooling, ice water was added, and the reaction mixture was neutralized with acetic acid. The resultant solid was filtered and washed with water, and the obtained filter cake was partitioned between ethyl acetate and water. The organic layer was concentrated under reduced pressure, and the resulting solid was filtered and washed with a little of ethyl acetate to give title product **6** (480 mg, 55.1%) as a light brown solid; ^{1}H NMR (500 MHz, $CDCl_3$) δ 6.69 (s, 1 H, 4-H), 6.28 (s, 1 H, 7-H), 5.87 (s, 2 H, CH_2), 4.20 (brs, 2 H, NH_2); ^{13}C NMR (100 MHz, DMSO-d_6) δ 150.5, 147.5, 138.9, 116.3, 101.3, 96.8.

2-(4-(5,6-methylenedioxybenzo[*d*]thiazol-2-yl)-2-methylphenoxy)-2-methyl propanoic acid (7)

A stirred solution of compound **3** (79 mg, 0.36 mmol) and compound **6** (66 mg, 0.39 mmol) in *N*,*N*-DMF (1 mL) was heated at 80 °C in the presence of $Na_2S_2O_5$ (81 mg, 0.43 mmol) for 20 h. DMF was evaporated under reduced pressure, and the residue was partitioned between ethyl acetate and water. The organic layer was dried over $MgSO_4$, filtered, and evaporated under reduced pressure. The crude product was purified by silica gel

column chromatography using methylene chloride and methanol (13:1) as the eluent to give title product **7** (43.5 mg, 32.9%) as a brown solid; melting point, 217.3–218.7 °C; ^{1}H NMR (500 MHz, DMSO-d_6) δ 7.81 (d, 1 H, J = 2.0 Hz, 3′-H), 7.72 (dd, 1 H, J = 2.0, 8.5 Hz, 5′-H), 7.61 (s, 1 H, 4″-H), 7.49 (s, 1 H, 7″-H), 6.78 (d, 1 H, J = 8.5 Hz, 6′-H), 6.11 (s, 2 H, CH_2), 2.23 (s, 3 H, 2′-CH_3), 1.57 (s, 6 H, 3-H_3, 2-CH_3); ^{13}C NMR (100 MHz, DMSO-d_6) δ 175.5, 166.0, 156.5, 149.3, 148.3, 147.0, 129.6, 129.6, 128.0, 126.6, 125.9, 116.2, 102.6, 102.5, 101.6, 79.4, 25.8, 17.0; HRMS (ESI) *m/z* $C_{19}H_{16}NO_5S$ (M-H)$^-$ calcd 370.0749, obsd370.0752.

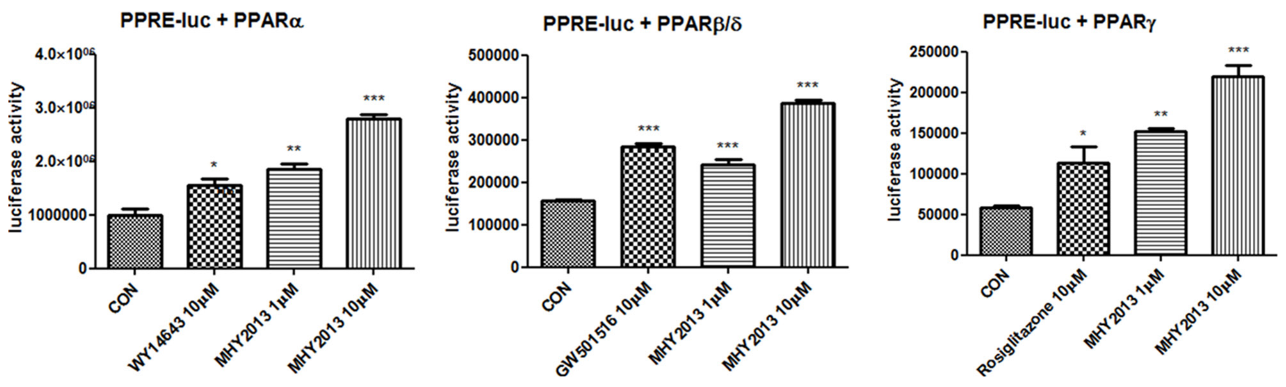

Supplementary Figure 1: MHY2013 dose-dependently increases transcriptional activity of three PPAR subtypes. Transcriptional activity of PPARα, PPARβ/δ, and PPARγ was dose-dependently increased by MHY2013. The data are shown as the mean ± SEM (n = 4). *, $p < 0.05$ vs. control (CON); **, $p < 0.01$ vs. CON; ***, $p < 0.001$ vs. CON.

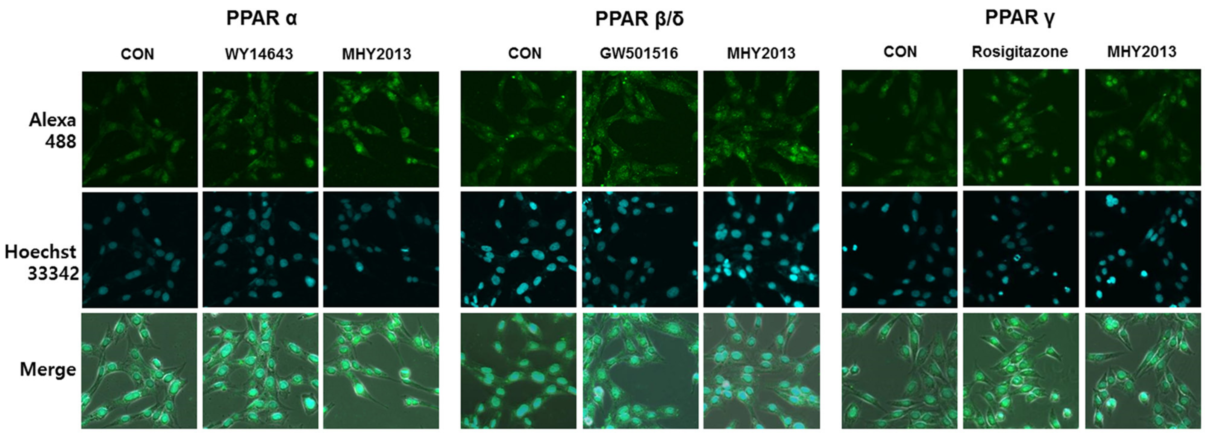

Supplementary Figure 2: MHY2013 induces translocation of three PPAR subtypes to the nucleus. YPEN-1 cells were treated with DMSO control (CON), respective PPAR agonists (WY14643, GW501516 or rosiglitazone) or MHY2013 for 90 min, fixed, permeabilized, and incubated with rabbit anti-PPARα, anti-PPARβ/δ, and anti-PPARγ primary antibodies, followed by incubation with a secondary anti-rabbit IgG antibody labeled with Alexa Fluor-488 (green). Nuclei were visualized by Hoechst 33342 staining (blue). Magnification, 60×.

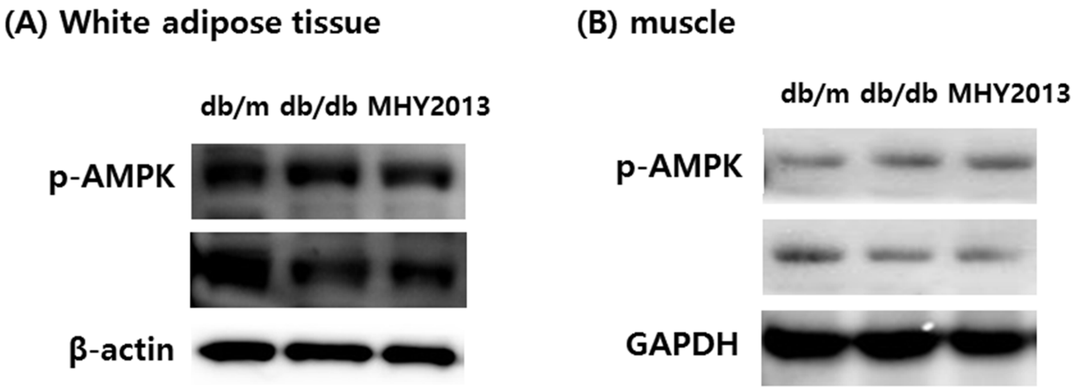

Supplementary Figure 3: The activation of AMPK in WAT and muscle is not changed by MHY2013 treatment. The mice were orally treated with the vehicle (water) or 5 mg/kg/day of MHY2013 for three weeks (n = 5). Activated p-AMPK (Thr172) was analyzed in **A.** WAT and **B.** muscle tissue by western blotting. β -Actin and GAPDH were used as the loading control.

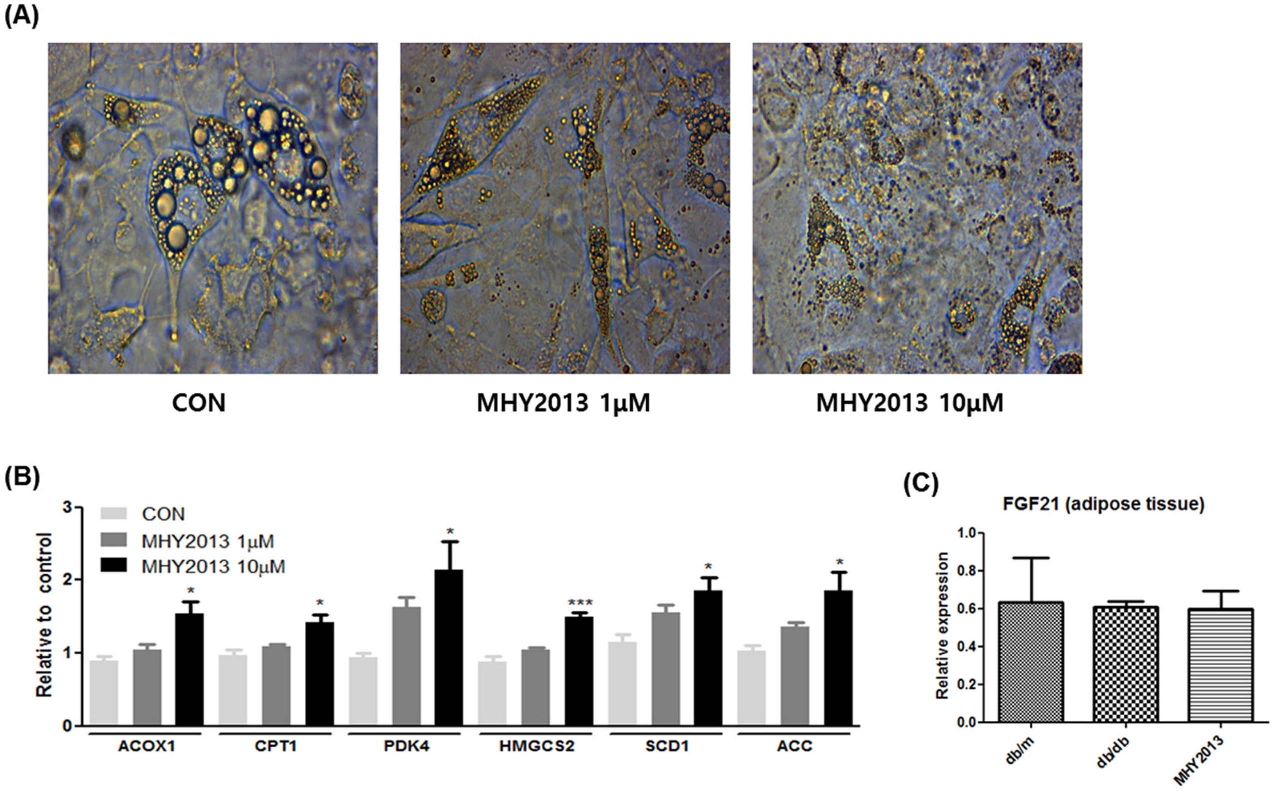

Supplementary Figure 4: MHY2013 increases the proportion of multi-locular lipid droplet structures. 3T3-L1 pre-adipocytes were differentiated into adipocytes for seven days with or without 10 μM MHY2013. **A.** An image of cellular morphology and lipid droplets. **B.** mRNA expression levels of fatty acid oxidation-related genes (*ACOX1*, *CPT1*, *HMGCS2*, and *PDK4*) were analyzed by qRT–PCR. *, $p < 0.05$ vs. control (CON); **, $p < 0.01$ vs. CON; ***, $p < 0.001$ vs. CON. The mice were orally treated with the vehicle (water) or 5 mg/kg/day of MHY2013 for three weeks (n = 5). **C.** In adipose tissue, mRNA expression levels of FGF21 were analyzed by qRT–PCR.

Supplementary Table 1: Substitution Pattern of the Substituted 2-methyl-2-phenoxypropanoic acid Derivatives

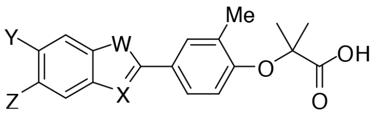

Compound	W	X	Y	Z
**MHY2013**	S	N	OCH_2O	
**MHY1907**	NH	N	H	H
**MHY2014**	NH	N	COOH	H
**MHY2015**	NH	N	Me	H
**MHY2016**	NH	N	COPh	H
**MHY2062**	S	N	OPh	H

Supplementary Table 2: Primer Sequences.

See Supplementary File: 1
